# Be Aware of Aggregators in the Search for Potential Human *ecto*-5′-Nucleotidase Inhibitors

**DOI:** 10.3390/molecules23081876

**Published:** 2018-07-27

**Authors:** Lucas G. Viviani, Erika Piccirillo, Arquimedes Cheffer, Leandro de Rezende, Henning Ulrich, Ana Maria Carmona-Ribeiro, Antonia T.-do Amaral

**Affiliations:** 1 Departamento de Química Fundamental, Instituto de Química, Universidade de São Paulo, Av. Prof. Lineu Prestes, 748, São Paulo 05508-000, Brazil; lucas.viviani@usp.br (L.G.V.); erika@iq.usp.br (E.P.); lrezende@iq.usp.br (L.d.R.); 2 Departamento de Bioquímica, Instituto de Química, Universidade de São Paulo, Av. Prof. Lineu Prestes, 748, São Paulo 05508-000, Brazil; arquiqbq@iq.usp.br (A.C.); henning@iq.usp.br (H.U.); mcribeir@iq.usp.br (A.M.C.-R.)

**Keywords:** aggregation, promiscuous mechanism, human *ecto*-5′-nucleotidase, virtual screening, enzymatic assays, turbidimetry, dynamic light scattering

## Abstract

Promiscuous inhibition due to aggregate formation has been recognized as a major concern in drug discovery campaigns. Here, we report some aggregators identified in a virtual screening (VS) protocol to search for inhibitors of human *ecto*-5′-nucleotidase (*ecto*-5′-NT/CD73), a promising target for several diseases and pathophysiological events, including cancer, inflammation and autoimmune diseases. Four compounds (**A**, **B**, **C** and **D**), selected from the ZINC-11 database, showed IC_50_ values in the micromolar range, being at the same time computationally predicted as potential aggregators. To confirm if they inhibit human *ecto*-5′-NT *via* promiscuous mechanism, forming aggregates, enzymatic assays were done in the presence of 0.01% (*v*/*v*) Triton X-100 and an increase in the enzyme concentration by 10-fold. Under both experimental conditions, these four compounds showed a significant decrease in their inhibitory activities. To corroborate these findings, turbidimetric assays were performed, confirming that they form aggregate species. Additionally, aggregation kinetic studies were done by dynamic light scattering (DLS) for compound **C**. None of the identified aggregators has been previously reported in the literature. For the first time, aggregation and promiscuous inhibition issues were systematically studied and evaluated for compounds selected by VS as potential inhibitors for human *ecto*-5′-NT. Together, our results reinforce the importance of accounting for potential false-positive hits acting by aggregation in drug discovery campaigns to avoid misleading assay results.

## 1. Introduction

Virtual screening (VS) and high-throughput screening (HTS) approaches have been well established as the main techniques for identification of bioactive compounds as potential drug candidates from large chemical libraries [1,2,3,4], showing significant success rates. However, currently it is well recognized that many screened hits are further recognized as not truly actives against their specific biological targets [5,6,7,8]. These compounds, usually termed “false hits” or “false positives″, act by a variety of mechanisms, including covalent protein reactivity, redox cycling, absorbance and/or fluorescence assay interference, membrane disruption, metal complexation, decomposition in assay buffers and formation of aggregates [8,9,10]. Thus, their activities do not depend on specific interactions with a binding site on the corresponding target protein. Accordingly, most of them do not show any structure-biological function relationship [10].

Small molecule aggregation, leading to promiscuous inhibition, in particular, seems to be the major source of false-positive results in drug discovery campaigns [5,8]. Molecular aggregates are formed in solution at micromolar or submicromolar concentrations, inhibiting or activating proteins nonspecifically in vitro, mainly by adsorption to protein surfaces [11]. Therefore, compounds classified in the literature as “aggregators″ are usually not suitable as drug candidates and their early identification can contribute to save time and money in drug discovery projects [5,6,12].

In order to minimize the impact of this important issue in drug design, computational methods, based mainly on physical and structural properties, have been proposed to identify and predict potential aggregators [5,12,13,14]. Despite the relevance of these methods, they have had only limited applicability and success rates, since the formation of aggregates depends on many different factors, such as temperature, ionic strength and both inhibitor and target protein concentrations, being very difficult to predict [5,15]. For this reason, such computational models should not be used to filter out potential aggregators from screening libraries, but only to quickly identify compounds that are potentially able to aggregate [5].

Thus, it has been stressed in the literature that the use of experimental procedures is the best way to detect aggregate formation and promiscuous inhibition mechanism in drug discovery projects as early as possible, reducing the number of data reports based on these artifacts [5,6,8]. It has been established that a molecule can be classified as an aggregator when it meets two or more of the following experimental criteria [5,8,11]: (i) attenuated activity in the presence of small amounts of a nonionic detergent, such as 0.01% (*v*/*v*) Triton X-100 or 0.025% (*v*/*v*) Tween-80 [11]; (ii) formation of aggregate particles in dispersion as detected by DLS [16,17,18]; (iii) noncompetitive inhibition with high Hill slopes [19]; (iv) attenuated inhibition by increasing target concentration [7,20]; (v) detergent-dependent inhibition of a well-established “counter-screen enzyme” [21], such as AmpC β-lactamase, trypsin or malate dehydrogenase, which show high sensitivity to compound aggregation; (vi) for cell based-assays, decreased activity after centrifugation of the medium, since aggregate particles can be precipitated by centrifugation [22].

Despite the importance of using suitable experimental procedures for detecting aggregation in drug discovery campaigns, so far only a few studies have drawn attention to compounds that showed typical aggregation behavior [2,6,13,23,24,25]. In addition, in most examples, the promiscuous behavior of some designed inhibitors is investigated just after they have already been reported as promising hits by scientific journals [8].

Here, in order to address and stress the issues of false positives and promiscuous inhibition mechanism in drug discovery campaigns, we describe some promiscuous aggregator inhibitors identified in a VS search for potential inhibitors of human *ecto*-5′-nucleotidase (*ecto*-5′-NT, CD73). *Ecto*-5′-NT is a key-enzyme in purinergic signaling pathways [26], which catalyzes the hydrolysis of AMP into adenosine and phosphate, playing a major role in the control of extracellular adenosine concentrations. Human *ecto*-5′-NT has been recognized as a promising biological target for many diseases and pathophysiological events [27], including cancer [28,29,30,31,32], autoimmune diseases [33], infections [34,35,36], atherosclerosis [37,38], ischemia-reperfusion injury [39] and central nervous system disorders [40]. Additionally, human *ecto*-5′-NT expression and activity have been used as a prognostic factor for multiple cancer types [41]. Considering its importance for therapy, the screening for *ecto*-5′-NT inhibitors has become urgent. Although numerous studies describing *ecto*-5′-NT inhibitors have been published in the literature [42,43,44,45,46,47,48,49,50], the corresponding procedures and controls concerning compound aggregation have not been systematically described so far for this target enzyme.

In this study, we observed that four compounds, designed and selected by a VS procedure as specific inhibitors of human *ecto*-5′-NT, significantly lost their inhibitory activities in the presence of 0.01% (*v*/*v*) Triton X-100), as well as at a 10-fold enzyme concentration increase. To corroborate these enzymatic study results, turbidimetric assays were performed, strongly suggesting that all these compounds probably form aggregates. In addition, aggregation kinetic studies were done, for one of them, by dynamic light scattering (DLS). These observations suggest typical aggregate formation and reinforce the need to control artifactual inhibition in drug discovery campaigns.

## 2. Results and Discussion

**Figure 1:**
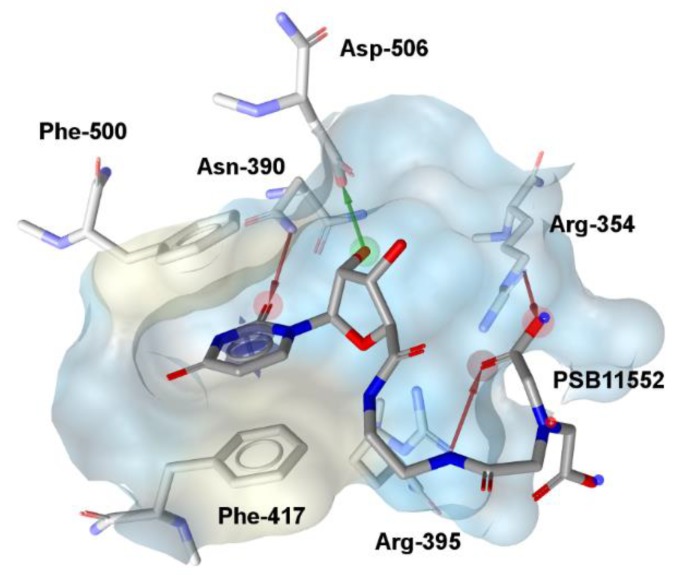
Pharmacophore model generated for PSB11552 complexed with human *ecto*-5′-NT, using LigandScout [51]. Green sphere: hydrogen-bond donor; red spheres: hydrogen-bond acceptors; blue circles: aromatic ring. The surface corresponding to PSB11552 binding site is colored according to lipophilic potential, ranging from white (highest lipophilic area surface) to cyan (highest hydrophilic area surface).

To search for novel potential human *ecto*-5′-NT inhibitors, a VS consisting of two consecutive filters (pharmacophore and docking complemented by visual inspection) was performed. Initially, a pharmacophore model was built, using LigandScout (Inte:Ligand, Maria Enzersdorf, Austria) [51], based on the 3D crystallographic structure of human *ecto*-5′-NT (in an open conformation) complexed with a peptidonucleoside inhibitor, PSB11552 (PDB code: 4H1Y) [52]. The generated pharmacophore model consists of five chemical features: one aromatic ring, one hydrogen bond donor and three hydrogen bond acceptors (Figure 1). Exclusion volume spheres were also considered, mimicking the cavity environment.

The pharmacophore model was applied to the ZINC-11 database (~23 × 10^6^ compounds) [53], from which 58 compounds matched all pharmacophore features. All of them were submitted to docking into the inhibitor binding site, using ChemPLP scoring function [54], available in GOLD. Subsequently, the best scored docking pose of each compound was submitted to visual inspection. In this last step, the following criteria were considered: (1) observation of mutual surface complementarity between ligand and protein; (2) presence of interactions with key-residues of the inhibitor binding site, specially π-stacking interactions with Phe-500 and Phe-417 side chains; hydrogen-bonds with backbone and/or side chain atoms from Asn-390, Asp-506, Arg-354 and Arg-395; hydrophobic interactions with Phe-500 and Phe-417; cation-π interactions with Arg-354 and Arg-395; (3) presence of additional interactions with residues located near the inhibitor binding site (e.g., hydrophobic interactions with Leu-415, Phe-421, Leu-389 and Thr-446 side chains); and (4) quality of the overall binding conformation to avoid clearly constrained conformations.

**Figure 2:**
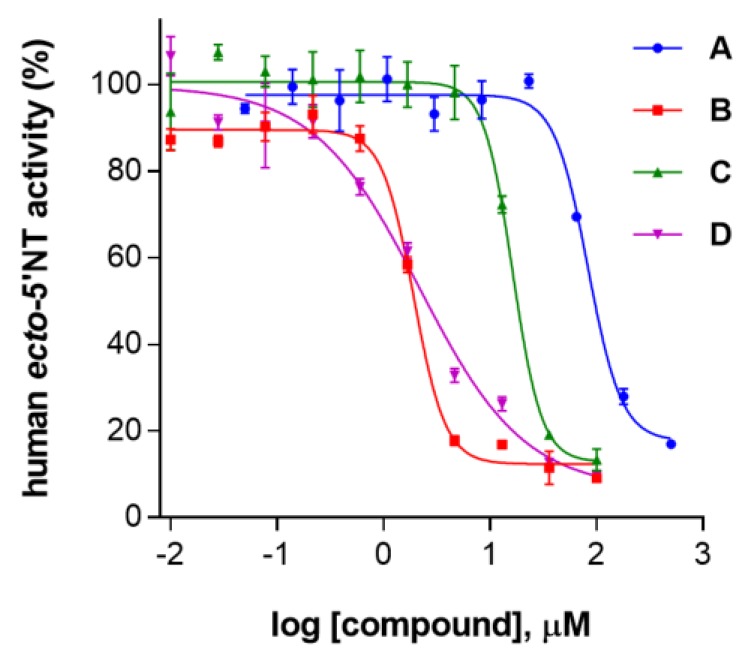
Dose-response curves for each tested compound (**A**, **B**, **C** and **D**). All assays were carried out in a reaction mixture containing HEPES buffer (10 mM; pH = 7.4), MgCl_2_ (2 mM), CaCl_2_ (1 mM), human *ecto*-5′-NT (3.6 nM), AMP (500 µM) as substrate, and each tested compound over a range of concentration values (0–500 µM for **A** and 0–100 µM for **B**, **C** and **D**). The concentration of DMSO in all samples was kept at 1.0% (*v*/*v*). After incubation for 10 min at 37.0 ± 0.2 °C, the reactions were stopped by heating the system for 5 min at 99.0 ± 0.2 °C. Inorganic phosphate released in the reaction was quantified spectrophotometrically (at λ = 630 nm), using the malachite green method, as described in the literature [56]. Data are expressed as the percentage of human *ecto*-5′-NT activity. Each experiment was done in triplicate. A four-parameter logistic non-linear regression model was used to fit the experimental data, using GraphPad Prism (GraphPad, San Diego, CA, USA).

Finally, 12 compounds, which met these visual inspection criteria, were selected as potential human *ecto*-5′-NT inhibitors, from which six were purchased and tested by enzymatic inhibition assays for VS experimental validation. Among the tested compounds, four showed IC_50_ values in the micromolar range (compounds **A**, **B**, **C** and **D**; Table 1) and two showed no significant inhibitory activity until *c.a.* 100 µM (i.e., less than 25% inhibition). The corresponding concentration-inhibition/dose-response curves are shown in Figure 2.

Although four compounds have shown at least moderate inhibitory activities against human *ecto*-5′-NT, one should note that steep concentration-inhibition curves were obtained for **A**, **B** and **C** (Hill slope values of −2.75, −2.90 and −3.19, respectively). For these three compounds, it is observed a sharp transition to almost full inhibition over a narrow range of concentrations (Figure 2). It is described that one possible interpretation for concentration-inhibition curves steepness is inhibition due to aggregation [19]. Additionally, compounds **B**, **C** and **D** have fairly high cLogP values (>3.0; see Table 1), which has also been recognized to be a typical physical chemical feature of aggregate-forming compounds [5].

Thus, to initially verify if the identified inhibitors are prone to aggregate, we used Aggregator Advisor tool (online available at *http://advisor.bkslab.org/*; provided by Shoichet Laboratory, UCSF, San Francisco, CA, USA) [5], which helps to distinguish between true and artifactual screening hits, based on Tanimoto structural similarity index (compared to known aggregators) and on lipophilicity criteria (based on calculated LogP). According to Aggregation Advisor predictions, **A**, **B** and **D** show high structural similarity with aggregators previously reported in the literature, as can be confirmed by their calculated Tanimoto index values (Table 2). Using the same similarity index, compound **C** did not show any structural similarity with aggregators comprised in the Aggregator Advisor database, but was also flagged as a potential aggregator, probably due to its high calculated Log P value (~3.6).

These computational predictions findings led us to use experimental controls to further investigate if compounds **A**, **B**, **C** and **D** are truly specific human *ecto*-5′-NT inhibitors or if they in fact act via aggregation. With this purpose, two experiments were initially performed, as suggested in the literature [5,6,8,11]: (i) enzymatic inhibition assays using a nonionic detergent (0.01% (*v/v*) Triton X-100) and (ii) enzymatic inhibition assays with a 10-fold increase in enzyme concentration.

**Figure 3:**
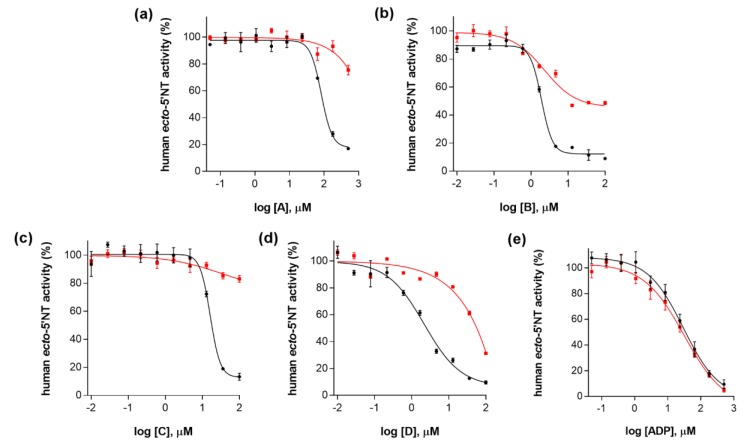
Dose-response curves for (**a**) compound **A**; (**b**) compound **B**; (**c**) compound **C**; (**d**) compound **D** and (**e**) ADP (negative control), without 0.01% (*v*/*v*) Triton X-100 (curves in black) and with 0.01% (*v*/*v*) Triton X-100 (curves in red). All assays were carried out in a reaction mixture containing HEPES buffer (10 mM; pH = 7.4), MgCl_2_ (2 mM), CaCl_2_ (1 mM), human *ecto*-5′-NT (3.6 nM), AMP (500 µM) as substrate, and tested compound over a range of concentration values (0–500 µM for **A** and ADP; and 0–100 µM for **B**, **C** and **D**), with or without 0.01% (*v*/*v*) Triton X-100. After incubation for 10 min at 37.0 ± 0.2 °C, the reactions were stopped by heating the system for 5 min at 99.0 ± 0.2 °C. Inorganic phosphate released in the reaction was quantified spectrophotometrically (at λ = 630 nm), using the malachite green method, as described in the literature [56]. For compounds **A**–**D**, the concentration of DMSO in all samples was kept at 1.0% (*v*/*v*). Data are expressed as the percentage of human *ecto*-5′-NT activity. Each experiment was done in triplicate. A four-parameter logistic non-linear regression model was used to fit the experimental data, using GraphPad Prism (GraphPad, San Diego, CA, USA).

Inhibitory activities of compounds **A** and **C** were almost fully reversed by Triton X-100 addition (Figure 3a,c), as attested by the increase in their corresponding IC_50_ values (from 82.9 ± 1.1 µM to >500 µM for **A** and from 16.3 ± 1.1 µM for >100 µM for **C**). Compounds **B** and **D** had their inhibitory activities partially lost when Triton X-100 was added in the assays (Figure 3b,d), as also can be verified by the increase in their corresponding IC_50_ values (from 1.9 ± 1.0 µM to 2.3 ± 1.2 µM for **B** and from 2.2 ± 1.2 µM to > *c.a.* 36 µM for **D**). Additionally, it should be emphasized that IC_50_ value calculated for **B** in the presence of Triton X-100 (0.01% (*v*/*v*)) is probably underestimated, since the minimum plateau value from its dose-response curve is far from zero, which means that full inhibition was not achieved for this compound (Figure 3b). It is important to report that adenosine diphosphate (ADP), known to be a specific, competitive and well-behaved inhibitor of mammalian *ecto*-5′-NT [57,58], was used as a negative control for aggregation studies. As expected, addition of detergent did not significantly affect ADP inhibitory activity against human *ecto*-5′-NT (Figure 3e), as attested by the IC_50_ values obtained in the absence (29.7 ± 1.2 µM) and in the presence (31.7 ± 1.2 µM) of 0.01% (*v*/*v*) Triton X-100).

These results suggest that the inhibitory activities of compounds **A**, **B**, **C** and **D** can be attributed, at least in part, to aggregate formation. According to the aggregation model proposed for protein inhibition, when an aggregate specie is formed in solution, proteins adsorb to its surface, being partially denatured, which leads to nonspecific inhibition [5,11]. Addition of a non-ionic detergent, such as Triton X-100, can disrupt the aggregates, leading to inhibitory activity loss [5,6,11].

**Figure 4:**
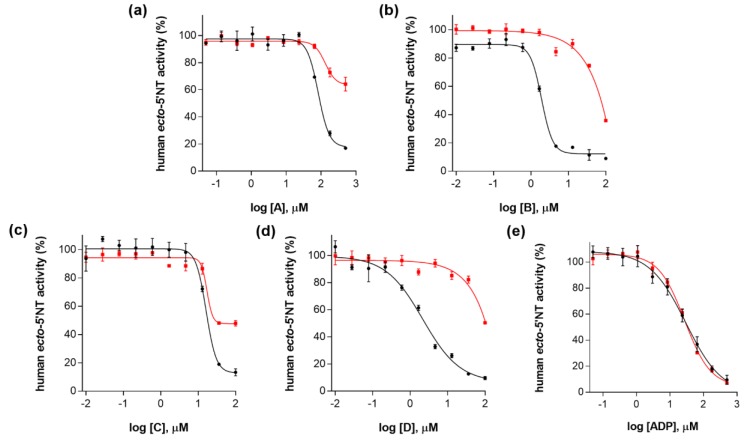
Dose-response curves for (**a**) compound **A**; (**b**) compound **B**; (**c**) compound **C**; (**d**) compound **D** and (**e**) ADP (negative control), at 3.6 nM (curves in black) and at 36 nM human *ecto*-5′-NT (curves in red). All assays were carried out in a reaction mixture containing HEPES buffer (10 mM; pH = 7.4), MgCl_2_ (2 mM), CaCl_2_ (1 mM), human *ecto*-5′-NT (3.6 nM or 36 nM), AMP (500 µM) as substrate, each tested compound over a range of concentration values (0–500 µM for **A** and ADP; and 0–100 µM for **B**, **C** and **D**), with or without 0.01% (*v*/*v)* Triton X-100. After incubation for 10 min at 37.0 ± 0.2 °C, the reactions were stopped by heating the system for 5 min at 99.0 ± 0.2 °C. Inorganic phosphate released in the reaction was quantified spectrophotometrically (at λ = 630 nm), using the malachite green method, as described in the literature [56]. For compounds **A**–**D**, the concentration of DMSO in all samples was kept at 1.0% (*v*/*v*). Data are expressed as the percentage of human *ecto*-5′-NT activity. Each experiment was done in triplicate. A four-parameter logistic non-linear regression model was used to fit the experimental data, using GraphPad Prism 7.0 (GraphPad, San Diego, CA, USA).

In agreement with our results obtained using 0.01% (*v*/*v*) Triton X-100, inhibitory activities of compounds **A**, **B**, **C** and **D** were, at least, partially lost when human *ecto*-5′-NT concentration was increased by 10-fold (i.e., from 3.6 nM to 36 nM). For compound **B**, IC_50_ value has increased from 1.9 ± 1.0 µM (at 3.6 nM *ecto*-5′-NT) to > *c.a.* 36 µM (at 36 nM *ecto*-5′-NT). Compound **D** had its IC_50_ value increased from 2.2 ± 1.2 µM (at 3.6 nM *ecto*-5′-NT) to > *c.a.* 36 µM (at 36 nM *ecto*-5′-NT). The IC_50_ values for compounds **A** and **C** at 36 nM of human *ecto*-5′-NT could not be properly obtained, since the minimum plateau values from their corresponding dose-response curves are far from zero (Figure 4a,c, curves colored in red). Nevertheless, it is reasonable to consider that the inhibitory activity for these two compounds were also reduced, by comparing their corresponding dose-response curves obtained at 3.6 nM (colored in black) and at 36 nM of human *ecto*-5′-NT (colored in red) (Figure 4a,c).

The partial loss of inhibitory activity observed for compounds **A**–**D**, when enzyme concentration was increased from 3.6 nM to 36 nM, suggests inhibition due to aggregation. It is well known that enzyme concentration dependence is typically observed for aggregate-based inhibitors [11,20], since the molar ratio of aggregate particles to enzyme is much lower than the corresponding molar ratio of a well-behaved inhibitor to enzyme. Accordingly, a considerable increase (≥10-fold) in enzyme concentration easily overwhelms the ability of aggregate particles to inhibit enzymatic activity [11,20].

Not surprisingly, for the negative control (ADP), the IC_50_ value obtained when the concentration of the enzyme was increased by 10-fold (27.2 ± 1.1 µM) was comparable to that obtained using 3.6 nM *ecto*-5′-NT (29.7 ± 1.2 µM) (Figure 4e). This observation agrees with the assumption that even a 10-fold increase in human *ecto*-5′-NT concentration was not enough to significantly affect the free concentration of ADP, a well behaved competitive inhibitor, which was present at micromolar concentrations.

**Figure 5:**
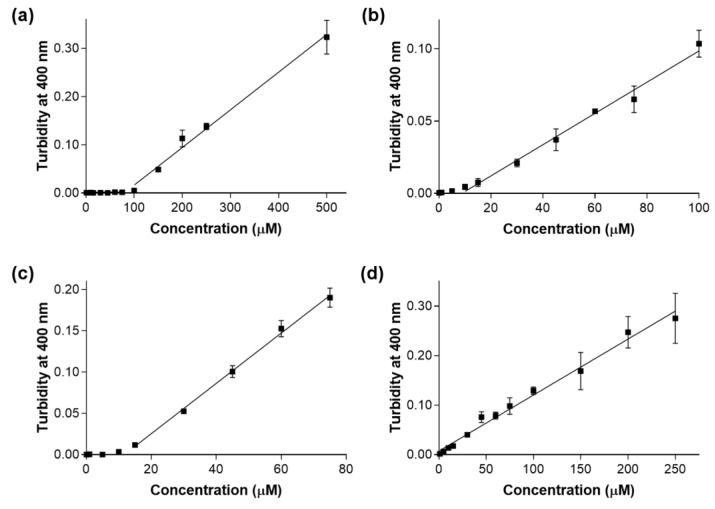
Turbidity at 400 nm as a function of concentration values measured for (**a**) compound **A**, (**b**) compound **B**, (**c**) compound **C** and (**d**) compound **D**. All solutions were prepared in HEPES buffer (10 mM, pH = 7.4) containing MgCl_2_ (2 mM) and CaCl_2_ (1 mM) salts. The final DMSO concentration in each sample was 1.0% (*v*/*v*). Each experiment was performed in triplicate.

To support our findings based on enzymatic assays, turbidimetric assays were done. As shown in Figure 5, from a critical concentration value, turbidity measured at 400 nm starts increasing, suggesting aggregation. This value corresponds to the estimated compound solubility in the assay buffer (Table 3). Interestingly, a reasonable correlation was observed between compound solubility and the corresponding predicted cLogP value for **A** and **D**. Compound **A**, which has the lower cLogP value (2.4), has the highest estimated solubility (79.1 µM). Compound **D**, in contrast, has been predicted to be the most lipophilic one (cLogP = 4.5) and shows the lowest estimated solubility (lower than 0.5 µM).

**Figure 6:**
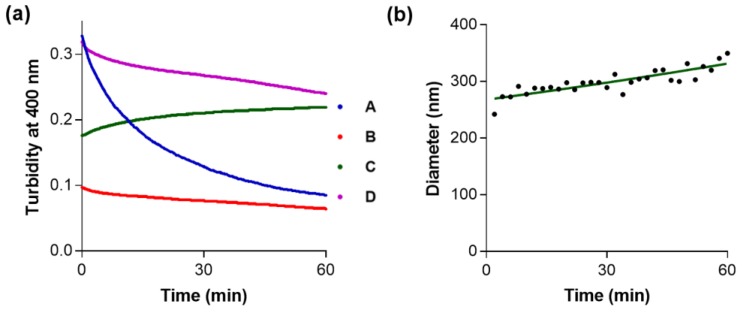
(**a**) Turbidity at 400 nm as a function of time measured for compounds **A**, **B**, **C** and **D** (at 500 µM, 100 µM, 80 µM and 250 µM, respectively). Each solution was prepared in HEPES buffer (10 mM), containing MgCl_2_ (2 mM) and CaCl_2_ (1 mM), pH = 7.4. Final concentration of DMSO in each sample was 1.0% (*v*/*v*); (**b**) Mean diameter (*D*) values as a function of time for compound **C** (80 µM) as determined by DLS. A solution of **C** was prepared in HEPES buffer (10 mM), containing MgCl_2_ (2 mM) and CaCl_2_ (1 mM), pH = 7.4. Final concentration of DMSO in each sample was 1.0% (*v*/*v*).

Additionally, turbidity at 400 nm as a function of time was followed for compounds **A**–**D** (Figure 6a). The concentration of each compound in these assays was near to the maximum that could be obtained, so that DMSO concentration was kept at 1.0% (*v*/*v*) in the assay buffer. For compounds **A**, **B** and **D**, a decrease in turbidity is observed as a function of time, in agreement with precipitation of these compounds verified in the assay buffer. In fact, after 60 min, precipitates at the bottom of the cuvettes were clearly observed by visual inspection (data not shown). Precipitation itself revealed rapid and massive aggregation with formation of heavy and large aggregates. For compound **C**, aggregate particle size slowly increased with time as shown by means of DLS (Figure 6b).

To expand our analysis concerning aggregation-based inhibition in the search for *ecto*-5′-NT inhibitors, we further analyzed 49 known *ecto*-5′-NT inhibitors described in the literature [42,43,44,46,52,57,58] to verify if they would be flagged as potential aggregators, using the Aggregator Advisor tool. These inhibitors were clustered considering: (i) structural similarity with compounds previously described as aggregators and (ii) calculated LogP values (Tables S1–S3, Supplementary Material). We observed that 12 of them (~25%), grouped as Cluster **1** (Table S1), were not flagged as potential aggregators since they are not structurally similar to any known aggregator and have calculated LogP values lower than 3.0. Cluster **2** (Table S2) includes 32 compounds (~65%), which are structurally similar to one aggregator from the database, but have calculated LogP values lower than 3. A critical analysis of the structures from this cluster reveals that the majority of them have a negatively charged or a polar group (compounds **LIT-13** to **LIT-43**, Table S2), which probably contributes to make them more hydrophilic. For this reason, they are likely not prone to aggregate. Alarmingly, however, one of the compounds from this cluster is quercetin, a well-known aggregator [2,13,21]. Cluster **3** (Table S3) comprises 5 compounds (~10%), which are not similar to previously described aggregators, but were appointed as possible aggregators due to their fairly high calculated LogP values (>3.0). Despite all these compounds contain a polar group in their structures, some of them have calculated LogP values up to 4.0. In summary, this preliminary analysis of known *ecto*-5′-NT inhibitors [42,43,44,46,52,57,58], using only a computational tool, warns the scientific community about the necessity to perform further experimental assays, in a systematic way, to discard the possibility of false-positive results among the human *ecto*-5′-NT inhibitors already described in the literature [42,43,44,46,52,57,58].

Taken together, the results obtained in our study suggest that the inhibitory activity of compounds **A**, **B**, **C** and **D**, selected by a VS protocol as potential human *ecto*-5′-NT inhibitors, can be explained, at least in part, by aggregation taking place over a range of micromolar concentrations. Thus, most likely these compounds are false-positive and promiscuous hits, which inhibit human *ecto*-5′-NT nonspecifically. To the best of our knowledge, they have not been previously reported as aggregators in the literature. One should notice that compound **C** was not shown to be significantly structurally similar to any other compound from the Aggregator Advisor tool database, despite its similarity with compounds **B** and **D** (Tanimoto similarity index values of 62% and 63%, respectively), which were recognized to be structurally similar to an aggregator from Aggregator Advisor (Table 2). These observations reinforce that computational methods to “advise″ aggregation are constantly under development and should always be complemented by experimental procedures. Additionally, compounds **A**, **B** and **D** themselves are not reported as aggregators in Aggregator Advisor, despite their relatively high Tanimoto similarity index values in relation to previously reported aggregators (Table 2). In this respect, this study provides novel data and information to feed Aggregator Advisor tool as well as other knowledge-based devices, thus contributing to increase the prediction power of such computational methods, which have been continuously refined over time.

For the first time, we describe aggregators identified on a VS search for human *ecto*-5′-NT. Due to its key role in purinergic signaling pathways regulation, *ecto*-5′-NT has been recognized as a promising biological target for multiple diseases and pathophysiological events, including cancer, autoimmune diseases, inflammation, infections and ischemia-reperfusion injury. The remarkable efforts that have been made by scientific community towards discovery of novel *ecto*-5′-NT inhibitors can be attested by the numerous studies that account for potential bioactive compounds and/or drug candidates targeting this enzyme [42,43,44,45,46,47,49,59]. Despite the encouraging results obtained by most of them, controls for inhibitors aggregation and/or precipitation have not been systematically reported so far.

Finally, our study reinforces the importance of performing accurate experimental procedures to control for aggregation as a fundamental step in experimental validation of VS results. Although it has been well accepted in the drug discovery community that identifying artifactual inhibition due to aggregation as early as possible is essential to save time and money, just a few studies have directly addressed this issue.

## 3. Materials and Methods

**Materials.** Purified recombinant human *ecto*-5′-nucleotidase was obtained from OriGene Technologies, Inc (Rockville, MD, USA); adenosine monophosphate (≥99%), adenosine diphosphate (≥99%), calcium chloride dihydrate (≥99%) and Triton X-100 were obtained from Sigma Aldrich, Inc (St. Louis, MO, USA); compound **A** ([(2,6-difluorophenyl)carbamoyl]methyl 1*H*-indazole-3-carboxylate) was obtained from Enamine Ltd (Kiev, Ukraine); compound **B** (*N*-(6-fluoro-1,3-benzothiazol-2-yl)-3-(2-hydroxyphenyl)-1*H*-pyrazole-5-carboxamide) was obtained from Pharmex, Ltd (Moscow, Russia); compound **C** (3-(2-hydroxy-3,5-dimethylphenyl)-*N*-[5-(methylsulfanyl)-1,3,4-thiadiazol-2-yl]-1*H*-pyrazole-5-carboxamide) and compound **D** (5-(2-hydroxyphenyl)-*N*-(6-methanesulfonyl-1,3-benzothiazol-2-yl)-1*H*-pyrazole-3-carboxamide) were obtained from Vitas-M Laboratory, Ltd, (Champaign, IL, USA); HEPES (2-[4-(2-hydroxyethyl)piperazin-1-yl]ethanesulfonic acid) (high purity grade) was obtained from Amresco, Inc (Solon, OH, USA); magnesium chloride anhydrous (≥99.9%) was obtained from USBiological Life Sciences, Co (Salem, MA, USA); green malachite oxalate, ammonium molybdate tetrahydrate (99%) and polyvinyl alcohol 98–99% hydrolyzed, high molecular weight, were obtained from Alfa Aesar, Co (Tewksbury, MA, USA); dimethyl sulfoxide (DMSO) was obtained from Merck, KGaA (Darmstadt, Germany).

Turbidimetric assays were done using a Hitachi U-2010 spectrophotometer (Hitachi, Chiyoda, Tokyo, Japan). DLS analysis was done using a Zeta Plus Zeta-Potential Analyzer (Brookshaven Instruments Corporation, Hotsville, NY, USA) equipped with a 570 nm laser for dynamic light scattering at 90°. For enzymatic assays, absorbance measurements were done using a FlexStation 3 Multi-Mode Microplate Reader (Molecular Devices, LLC, San Jose, CA, USA).

Aggregator Advisor tool (available online on *http://advisor.bkslab.org/*; provided by Shoichet Laboratory, UCSF, San Francisco, CA, USA) [5] was used to predict potential aggregators.

ZINC-11 database (~23 × 10^6^ compounds) [53] was used for virtual screening.

**Virtual screening.** In a first step, a pharmacophore model (generated using the LigandScout 4.1 program, Inte:Ligand GmbH, Maria Enzersdorf, Austria; *www.inteligand.com*) [51], based on the available crystallographic 3D structure of human *ecto*-5′-NT complexed with a peptidonucleoside inhibitor (PSB11552) (PDB code: 4H1Y) [52], was generated and applied to the ZINC-11 database (conformers generated by OMEGA 2.4.3 program, OpenEye Scientific Software, Santa Fe, NM, USA) [60]). H-bond acceptor and donor features have 1.95 Å tolerance radius and the aromatic ring feature has 0.90 Å tolerance radius. Exclusion volume spheres were created based on the binding-site residues positions. Subsequently, compounds from ZINC-11 that matched all pharmacophore features were docked into human *ecto*-5′-NT adenosine binding site (in *ecto*-5′-NT open conformation), using GOLD 5.2 (CCDC, Cambridge, UK) [61], scoring function ChemPLP [54]. The binding site was defined as a sphere with 10 Å radius, centered at *X* = 13.817; *Y* = 11.61 and *Z* = 37.81. In all docking calculations, GOLD default settings were applied, using the maximum search efficiency. For each compound, 10 docking runs were performed. Finally, the best pose of each docked compound was subjected to a visual inspection and those that best fitted into adenosine binding site were selected as potential *ecto*-5′-NT inhibitors.

**LogP values calculation.** cLogP (n-octanol/water as partition model system) values were obtained with LigandScout 4.01 [51], using the topological cLogP estimation algorithm of Wildman and Crippen [55].

**Tanimoto index values calculation.** Instant JChem was used for calculating the Tanimoto values between compounds **C** and **B** and **C** and **D** applying the default Chemical Hashed Fingerprint, Instant JChem 18.13.0, ChemAxon (Budapest, Hungary) (https://www.chemaxon.com).

**Enzyme inhibition assays (without Triton X-100).** Following procedures described in the literature [62], with some modifications, all assays were carried out in a reaction mixture containing HEPES buffer (10 mM; pH = 7.4), MgCl_2_ (2 mM), CaCl_2_ (1 mM), human *ecto*-5′-NT (3.6 nM), AMP (500 µM) as substrate and variable concentration of each tested compound (from 0 to 500 µM for **A** and from 0 to 100 µM for **B**, **C** and **D**). Stock solutions of each compound were prepared in DMSO. The final concentration of DMSO in all samples/assays/experiments was 1.0% (*v*/*v*). Results were controlled for the effect of DMSO on enzymatic activity. After incubation for 10 min at 37.0 ± 0.2 °C, the reactions were stopped by heating the system for 5 min at 99.0 ± 0.2 °C. Inorganic phosphate concentrations were quantified spectrophotometrically (at λ = 630 nm), using the malachite green method, as described in the literature [56]. Each experiment was done in triplicate. A four-parameter logistic non-linear regression model was used to fit the experimental data, using GraphPad Prism 7.0 (GraphPad, San Diego, CA, USA). From the corresponding fitted curves, we obtained the IC_50_ values, except when the minimum plateau value from the dose-response curve was far from zero. For such curves, IC_50_ ranges were estimated based on the inhibition (%) achieved at the maximum tested concentration.

**Promiscuous inhibition mechanism aggregation studies:** As proposed in the literature [5,8,11], promiscuous inhibition mechanism was analyzed through the following experiments:

(*i*) Non-ionic detergent-sensitivity evaluation: For each compound (**A**, **B**, **C** and **D**), enzyme inhibition assays were done, similarly as described above, using however Triton X-100 (a non-ionic detergent) at a final concentration of 0.01% (*v*/*v*) in the reaction mixture.

(*ii*) Enzyme concentration sensitivity evaluation: For each compound (**A**, **B**, **C** and **D**), enzyme inhibition assays were done, similarly as described above, but using human *ecto*-5′-NT at 36 nM (increased by 10-fold).

(*iii*) Turbidimetric solubility assays: Solutions of each compound (**A**, **B**, **C** and **D**) were prepared at multiple concentrations by diluting concentrated DMSO stock solutions into HEPES buffer (10 mM, pH = 7.4) containing MgCl_2_ (2 mM) and CaCl_2_ (1 mM) salts. The final DMSO concentration in each sample was 1.0% (*v*/*v*). Increased turbidity (light scattering) was measured at 400 nm, since all compounds have absorbance peaks below this wavelength. Each sample was prepared and measured in triplicate. All measurements were done using a Hitachi U-2010 spectrophotometer.

(*iv*) Dynamic light scattering (DLS): Particle size (mean zeta-average diameter *D*) for compound **C** was determined using a Zeta Plus Zeta-Potential Analyzer (Brookshaven Instruments Corporation, Hotsville, NY, USA) equipped with a 570 nm laser for dynamic light scattering at 90° [63]. Solutions of Compound **C** (80 µM) were prepared in HEPES buffer (10 mM), pH = 7.4. The final concentration of DMSO in each sample was 1.0% (*v*/*v*).

## 4. Conclusions

This study reports the identification of four false positive hits selected on a VS search for human *ecto*-5′-NT inhibitors. These compounds inhibited human *ecto*-5′-NT nonspecifically, most likely acting by aggregate formation, as suggested by computational predictions and confirmed by experimental procedures, including non-ionic detergent-based assays, evaluation of enzyme concentration effect on inhibitory activity, turbidimetric assays and, eventually, DLS experiments. To the best of our knowledge, none of the identified compounds has previously been reported as an aggregator in the literature. For the first time, the aggregation and promiscuous inhibition issues were systematically studied and evaluated for compounds selected as potential inhibitors of human *ecto*-5′-NT (CD73), an enzyme that has increasingly attracted attention of scientific community due to its potential as a biological target for many diseases and pathophysiological conditions, especially inflammation, immune imbalance and cancer.

Together, the results and data reported here reinforce the importance of performing accurate experimental procedures to identify aggregators, which are recognized as a major source of false-positives in drug discovery campaigns. Early identification of aggregate-forming compounds, acting by promiscuous mechanism, contributes to avoid misleading results, saving time and money in drug discovery projects.

## Figures and Tables

**Table 1:** Chemical structures, physical-chemical properties (molecular weight and cLogP values) and IC_50_ values obtained for four *ecto*-5′-NT inhibitors (**A**, **B**, **C** and **D**) selected by VS.

Compound (ID)	Structure	Molecular Weight (g·mol^−1^)	cLogP ^1^	IC_50_ (µM) ^2^
**A**	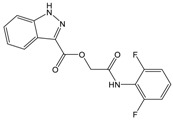	331.28	2.4	82.9 ± 1.1
**B**	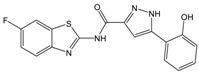	354.36	4.2	1.9 ± 1.0
**C**	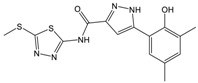	361.45	3.6	16.3 ± 1.1
**D**	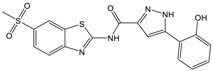	414.46	4.5	2.2 ± 1.2

^1^ Values calculated with LigandScout 4.1 [51], using the topological cLogP estimation algorithm of Wildman and Crippen [55]. ^2^ Values obtained from a four-parameter logistic nonlinear model used to fit the experimental data from dose-response curves (Figure 2). All experiments were performed in a reaction mixture containing HEPES buffer (10 mM; pH = 7.4), MgCl_2_ (2 mM), CaCl_2_ (1 mM), human *ecto*-5′-NT (3.6 nM), AMP (500 µM) as substrate and each tested compound over a range of concentration values (0–500 µM for **A** and 0–100 µM for **B**, **C** and **D**). The concentration of DMSO in all samples was kept at 1.0% (*v*/*v*). Inorganic phosphate released in the reaction was quantified spectrophotometrically (at λ = 630 nm), using the malachite green method, as described in the literature [56].

**Table 2:** Chemical structures of compounds **A**, **B**, **C** and **D**, chemical structures of some previously reported aggregators, and the corresponding Tanimoto similarity index values (%), obtained using Aggregator Advisor tool [5].

Compound (ID)	Structure	Previously Reported Aggregator (Structure)	Tanimoto Similarity Index Value (%) ^1^	Reference
**A**	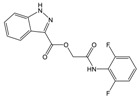	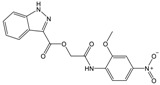	72	[2]
**B**	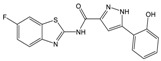	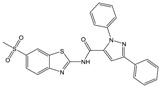	72	[2]
**C**	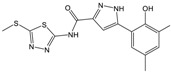	n.s.^2^		
**D**	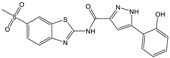	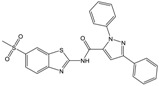	81	[2]

^1^ Values calculated using Aggregator Advisor Tool (online available at *http://advisor.bkslab.org/*) [5]. ^2^ n.s. means not similar to any compound from Aggregator Advisor database.

**Table 3:** cLogP and estimated solubility values for each compound (**A**, **B**, **C** and **D**).

Compound (ID)	cLogP ^1^	Estimated Solubility (µM) ^2^
**A**	2.4	79.1
**B**	4.2	8.8
**C**	3.6	11.7
**D**	4.5	< 0.5 *

^1^ Values calculated with LigandScout [51], using the topological cLogP estimation algorithm of Wildman and Crippen [55]. ^2^ Values calculated from turbidimetric solubility assays (Figure 5). * The estimated solubility could not be accurately calculated for compound **D**, due to method sensitivity limitations.

## Supplementary Materials

Supplementary materials are available online: Tables S1–S3.
