# Supplementary material for: Be Aware of Aggregators in the Search for Potential Human ecto-5′-Nucleotidase Inhibitors
Source: Molecules. 2018 Jul 27;23(8):1876. doi: 10.3390/molecules23081876 (PMC6222861; doi:10.3390/molecules23081876)
Supplement: Supplementary file 1 [file molecules-23-01876-s001.pdf]

Be Aware of Aggregators in the Search for Potential Human *ecto*-5′-nucleotidase Inhibitors

As described in the manuscript, 49 known *ecto*-5′-NT inhibitors (here named **LIT-01** to **LIT-49**) were grouped in three clusters, according to Aggregator Advisor tool [1] results:

- **Cluster 1 (Table S1)**: compounds that (i) were not found to be similar to any previously reported aggregator and (ii) have calculated LogP values lower than 3.
- **Cluster 2 (Table S2)**: compounds that (i) were found to be similar to one previously reported aggregator and (ii) have calculated LogP values lower than 3.
- **Cluster 3 (Table S3)**: compounds that (i) were not found to be similar to any previously reported aggregator and (ii) have calculated LogP values higher than 3.

Table S1. Chemical structures of compounds grouped as **Cluster 1** (**LIT-01** to **LIT-12**).

Compound (ID)	Structure	Ref.
**LIT-01**	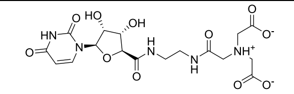	[2]
**LIT-02**	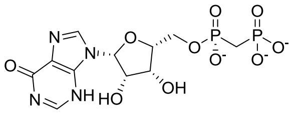	[3]
**LIT-03** to **LIT-05**	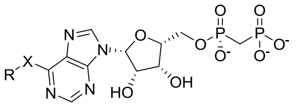<br>LIT-03: X=O and R = CH_2CH_3,<br>LIT-04: X=O and R = Bez[2]<br>LIT-05: X=S and R =Bez[2]	[3]
**LIT-)06**	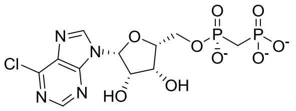	[3]
**LIT-07**	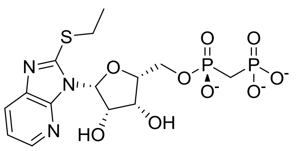	[3]
**LIT-08**	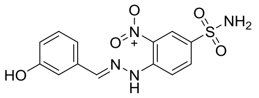	[4]
**LIT-09**	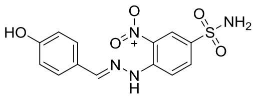	[4]

**LIT-10**	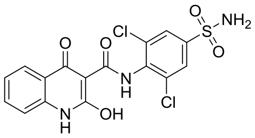	[4]
**LIT-11**	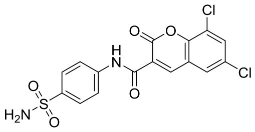	[4]
**LIT-12**	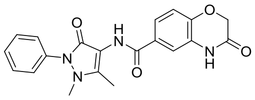	[4]

[1]Values calculated using Aggregator Advisor Tool (online available at *http://advisor.bkslab.org/*) [1].

[2]Bez: benzyl group

Table S2. Chemical structures of compounds grouped as **Cluster 2** (**LIT-13** to **LIT-44**), chemical structures of some previously reported aggregators, and the corresponding Tanimoto similarity index values (%), obtained using Aggregator Advisor tool.

Compound (ID)	Structure	Ref.	Previously reported aggregator (structure)	Ref.	Tanimoto similarity index value (%)[1]
**LIT-13**	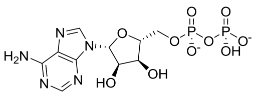	[5]	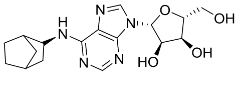	[6]	75
**LIT-14**	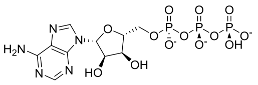	[7]	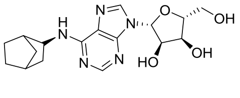	[6]	74
**LIT-15**	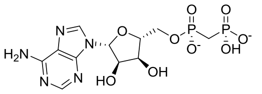	[2]	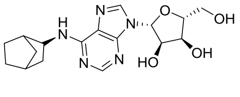	[6]	73
**LIT-16** to **LIT-23**	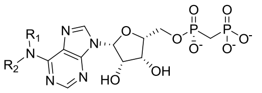<br>**LIT-16**: R_1 = H and R_2 = CH_3<br>**LIT-17**: R_1 = H and R_2 = CH_2CH_3<br>**LIT-18**: R_1 = R_2 = CH_3<br>**LIT-19**: R_1 = R_2 = CH_2CH_3<br>**LIT-20**: R_1 = CH_3 and R_2 = CH_2CH_3<br>**LIT-21**: R_1 = H and R_2 = Phe[2]<br>**LIT-22**: R_1 = CH_2CH_3 and R_2 = Bez[3]<br>**LIT-23**: R_1 = R_2 = Bez[3]	[3]	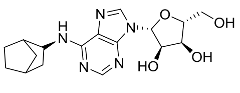	[6]	71 to 78
**LIT-24** to **LIT-28**	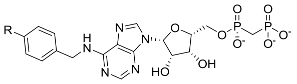<br>**LIT-24**: R = H<br>**LIT-25**: R = Cl,<br>**LIT-26**: R = NH_2<br>**LIT-27**: R = SO_2NH_2<br>**LIT-28**: R = NO_2	[3]	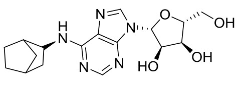	[6]	71 to 74

**LIT-29**	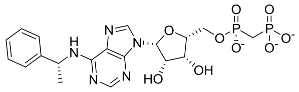	[3]	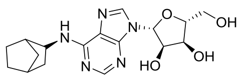	[6]	75
**LIT-30** to **LIT-33**	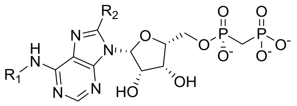<br>**LIT-30**: R_1 = H and R_2 = Cl<br>**LIT-31**: R_1 = H and R_2 = Br<br>**LIT-32**: R_1 = H and R_2 = $NHCH_3$<br>**LIT-33**: R_1 = Bez and R_2 = Br	[3]	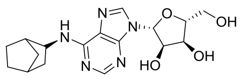	[6]	70 or 71
**LIT-34** to **LIT-36**	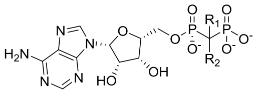<br>**LIT-34**: R_1 = R_2 = Cl<br>**LIT-35**: R_1 = R_2 = Br<br>**LIT-36**: R_1 = CH_3 and R_2 = OH	[3]	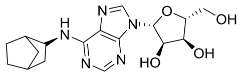	[6]	71
**LIT-37** to **LIT-40**	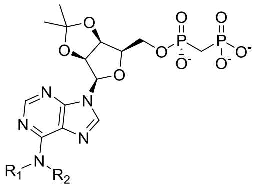<br>**LIT-37**: R_1 = H and R_2 = CH_3<br>**LIT-38**: R_1 = H and R_2 = CH_2CH_3<br>**LIT-39**: R_1 = R_2 = CH_3<br>**LIT-40**: R_1 = R_2 = CH_2CH_3	[3]	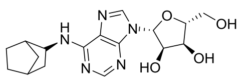	[6]	72 to 74
**LIT-41**	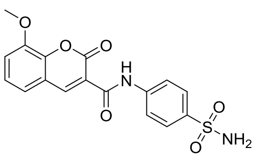	[4]	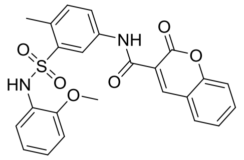	[6]	74
**LIT-42**	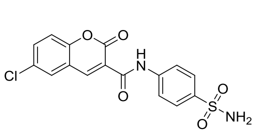	[4]	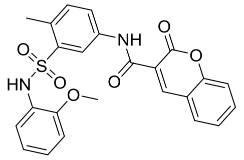	[6]	71
**LIT-43**	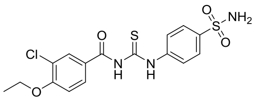	[4]	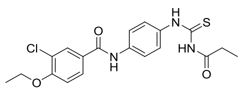	[6]	75
**LIT-44**	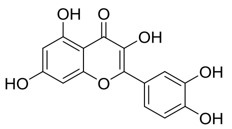	[8]	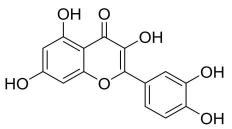		100

[1]Values calculated using Aggregator Advisor Tool (online available at *http://advisor.bkslab.org/*) [1].

[2]Phe: phenyl group

[3]Bez: benzyl group

Table S3. Chemical structures of compounds grouped as **Cluster** **3** (**LIT-45** to **LIT-49**), and their corresponding calculated LogP values, obtained using Aggregator Advisor tool.

Compound (ID)	Structure	Ref.	Calculated LogP value[1]
**LIT-45**	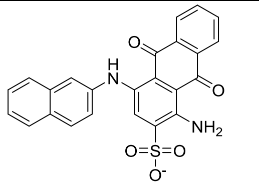	[9]	3.1
**LIT-46**	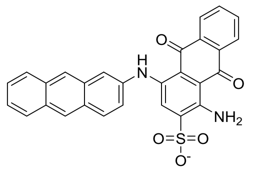	[9]	4.3
**LIT-47**	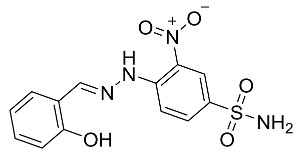	[4]	3.3
**LIT-48**	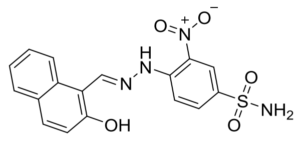	[4]	4.4
**LIT-49**	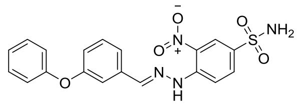	[4]	5.1

[1]Values calculated using Aggregator Advisor Tool (online available at *http://advisor.bkslab.org/*) [1].

References

1. Irwin, J. J.; Duan, D.; Torosyan, H.; Doak, A. K.; Ziebart, K. T.; Sterling, T.; Tumanian, G.; Shoichet, B. K. An Aggregation Advisor for Ligand Discovery. *J. Med. Chem.* **2015**, *58*, 7076–7087, doi:10.1021/acs.jmedchem.5b01105.
2. Knapp, K.; Zebisch, M.; Pippel, J.; El-Tayeb, A.; Müller, C. E.; Sträter, N. Crystal structure of the human ecto-5′-nucleotidase (CD73): Insights into the regulation of purinergic signaling. *Structure* **2012**, *20*, 2161–2173, doi:10.1016/j.str.2012.10.001.
3. Bhattarai, S.; Freundlieb, M.; Pippel, J.; Meyer, A.; Abdelrahman, A.; Fiene, A.; Lee, S.-Y.; Zimmermann, H.; Yegutkin, G. G.; Sträter, N.; El-Tayeb, A.; Müller, C. E. *α*,β-Methylene-ADP (AOPCP) Derivatives and Analogues: Development of Potent and Selective ecto -5′-Nucleotidase (CD73) Inhibitors. *J. Med. Chem.* **2015**, *58*, 6248–6263, doi:10.1021/acs.jmedchem.5b00802.
4. Ripphausen, P.; Freundlieb, M.; Brunschweiger, A.; Zimmermann, H.; Müller, C. E.; Bajorath, J. Virtual Screening Identifies Novel Sulfonamide Inhibitors of ecto -5′-Nucleotidase. *J. Med. Chem.* **2012**, *55*, 6576–6581, doi:10.1021/jm300658n.
5. Freundlieb, M.; Zimmermann, H.; Müller, C. E. A new, sensitive ecto-5???-nucleotidase assay for compound screening. *Anal. Biochem.* **2014**, *446*, 53–58, doi:10.1016/j.ab.2013.10.012.
6. Ferreira, R. S.; Simeonov, A.; Jadhav, A.; Eidam, O.; Mott, B. T.; Keiser, M. J.; McKerrow, J. H.; Maloney, D. J.; Irwin, J. J.; Shoichet, B. K. Complementarity between a docking and a high-throughput screen in discovering new cruzain inhibitors. *J. Med. Chem.* **2010**, *53*, 4891–4905, doi:10.1021/jm100488w.
7. Iqbal, J.; Jirovsky, D.; Lee, S. Y.; Zimmermann, H.; M??ller, C. E. Capillary electrophoresis-based nanoscale assays for monitoring ecto-5???-nucleotidase activity and inhibition in preparations of

recombinant enzyme and melanoma cell membranes. *Anal. Biochem.* **2008**, *373*, 129–140, doi:10.1016/j.ab.2007.09.028.

8. Braganhol, E.; Tamajusuku, A. S. K.; Bernardi, A.; Wink, M. R.; Battastini, A. M. O. Ecto-5′-nucleotidase/CD73 inhibition by quercetin in the human U138MG glioma cell line. *Biochim. Biophys. Acta - Gen. Subj.* **2007**, *1770*, 1352–1359, doi:10.1016/j.bbagen.2007.06.003.
9. Baqi, Y.; Lee, S.; Iqbal, J.; Ripphausen, P.; Lehr, A.; Scheiff, A. B.; Zimmermann, H.; Bajorath, J.; Müller, C. E. Development of Potent and Selective Inhibitors of ecto -5′-Nucleotidase Based on an Anthraquinone Scaffold. *J. Med. Chem.* **2010**, *53*, 2076–2086, doi:10.1021/jm901851t.
